# Automated methods of predicting the function of biological sequences using GO and BLAST

**DOI:** 10.1186/1471-2105-6-272

**Published:** 2005-11-15

**Authors:** Craig E Jones, Ute Baumann, Alfred L Brown

**Affiliations:** 1 Australian Centre for Plant Functional Genomics, Waite Campus, University of Adelaide, South Australia, 5064, Australia; 2 School of Computer Science, University of Adelaide, South Australia, 5001, Australia

## Abstract

**Background:**

With the exponential increase in genomic sequence data there is a need to develop automated approaches to deducing the biological functions of novel sequences with high accuracy. Our aim is to demonstrate how accuracy benchmarking can be used in a decision-making process evaluating competing designs of biological function predictors. We utilise the Gene Ontology, GO, a directed acyclic graph of functional terms, to annotate sequences with functional information describing their biological context. Initially we examine the effect on accuracy scores of increasing the allowed distance between predicted and a test set of curator assigned terms. Next we evaluate several annotator methods using accuracy benchmarking. Given an unannotated sequence we use the Basic Local Alignment Search Tool, BLAST, to find similar sequences that have already been assigned GO terms by curators. A number of methods were developed that utilise terms associated with the best five matching sequences. These methods were compared against a benchmark method of simply using terms associated with the best BLAST-matched sequence (best BLAST approach).

**Results:**

The precision and recall of estimates increases rapidly as the amount of distance permitted between a predicted term and a correct term assignment increases. Accuracy benchmarking allows a comparison of annotation methods. A covering graph approach performs poorly, except where the term assignment rate is high. A term distance concordance approach has a similar accuracy to the best BLAST approach, demonstrating lower precision but higher recall. However, a discriminant function method has higher precision and recall than the best BLAST approach and other methods shown here.

**Conclusion:**

Allowing term predictions to be counted correct if closely related to a correct term decreases the reliability of the accuracy score. As such we recommend using accuracy measures that require exact matching of predicted terms with curator assigned terms. Furthermore, we conclude that competing designs of BLAST-based GO term annotators can be effectively compared using an accuracy benchmarking approach. The most accurate annotation method was developed using data mining techniques. As such we recommend that designers of term annotators utilise accuracy benchmarking and data mining to ensure newly developed annotators are of high quality.

## Background

Genomics research is generating enormous quantities of DNA and protein sequence data. GenBank, a major repository of genomic data, reports an exponential increase in sequence data, in the last 10 years the quantity of data has increased more than two-hundred-fold [1]. Sequence data alone is of limited use to biologists, being simply a linear array of base or amino acid codes. To make the most of the data biologists need to be able to place the sequence within a biological context, that is, they require information concerning the biological properties and functions that the DNA or protein sequence might be considered to have from an expert's point of view. This has led to the need for software able to conduct high-throughput, accurate function prediction.

The creation of the Gene Ontology, GO, has provided a rich resource for describing the functional characteristics of sequences [2]. GO is widely used, both in the analysis of microarray data and generally in comparative genomics, to abstract above the level of sequences to that of function. Essentially GO contains three ontologies, describing the biological process, cellular compartment and molecular function properties of sequences. Each ontology is a directed acyclic graph of functional term nodes where edges between nodes describe relationships between them. GO is now the defacto-standard for annotating sequences with functional information.

The Basic Local Alignment Search Tool, BLAST, is the most commonly used sequence alignment application [3,4]. It allows the user to find sequences with high degrees of local similarity to query sequences. Furthermore, it supports the creation of custom sequence databases. For these reasons BLAST has been employed to assign GO terms to novel sequences, the assumption being that GO terms belonging to similar sequences will have a high likelihood of also belonging to the query sequence.

BLAST assigns an expect value to each sequence found in the sequence database based on a local alignment between that sequence and the input sequence. The expect value is based on a score assigned to gapped alignments between sequences, the size of the database, and the lengths of both sequences. Expect values less than 0.01 can be considered to be the same as the probability that two sequences match purely by chance [5]. Therefore the lower the expect value the more significant the match between sequences.

Recently a number of accounts of automated BLAST-based GO term prediction applications have been published. GOblet [6,7] is a web-based system allowing users to find GO terms for Gene Ontology Annotation database [8] curated sequences. GOFigure [9] is also a web-based system that uses BLAST to find matching sequences with existing GO term annotations, and then constructs a minimum covering graph of term nodes. Terms are assigned a score based on the expect value of the matching sequence to which they were assigned. Parents are then assigned scores associated with their child nodes. GOEngine [10] utilises a variety of data sources from literature mining to BLAST homolog analysis. In addition, data-source oriented function annotation projects utilise BLAST as a way of predicting GO terms based on sequence similarity. Such projects include the Gene Ontology Annotation Database associated with the European Bioinformatics Institute [8], NetAffx [11] associated with Affymetrix microarray probe-sets, as well as species-specific investigations [12,13].

Most published accounts of automated approaches to BLAST-based GO term prediction have demonstrated their accuracy using ad hoc methods following implementation. It is our aim to demonstrate how well planned accuracy benchmarking can be used in a decision-making process evaluating competing designs – a useful first step towards producing a high accuracy biological function predictor. Furthermore, because of the fact that terms are united by an ontology, some researchers [9,10] have allowed terms within a given number of edges to be counted as correct. As our approach is heavily dependent on precision and recall measures, we examine the impact of increasing the allowed distance between correct and predicted term nodes on the reliability of these measures.

## Results

### Background

#### Data collection and preparation

The March 2004 Gene Ontology data [14] was downloaded and imported into a MySQL database. This data consists of both protein sequence data and their GO term associations. Only proteins and their GO term associations were included in this study if term annotations were made manually, i.e. did not have the GO evidence code [15] of inferred from sequence similarity or ISS.

The resulting "manually curated" protein term associations were broken into two distinct groups:

1. UniProt [16] annotations – proteins and their GO term associations that were submitted by UniProt. This data, consisting of 7071 proteins with high quality annotations, was referred to as the 'training set'.

2. Non UniProt annotations – proteins and their GO term associations that were submitted by FlyBase, Mouse Genome Informatics (MGI), Sanger GeneDB, Saccharomyces Genome Database, and The Institute for Genome Research (TIGR). This data was referred to as the 'test set'. It consists of 19965 annotated proteins, and can be assumed to have greater variation in annotation quality.

This data provided us with a set of known 'correct' annotations for proteins, and was used to assess the effectiveness of various 'annotation methods'. Generally speaking non-data-mining approaches used the 'training set' to assess their annotation accuracy, while data-mining approaches used the 'training set' for model creation, and the 'test set' for model assessment.

Two BLAST-able databases were created using NCBI-BLAST's formatdb command, one for the training and test sets respectively. Also, all protein sequences were written to individual fasta format text files to allow for BLAST searches where they were the query sequence.

#### Accuracy metrics

We modified definitions of precision and recall measures to be applicable to assessing the accuracy of term assignment predictions. Given that a protein sequence has a set of correct term associations, and that an annotation method will provide a set of predicted term associations, precision and recall were defined here as:

P = c/p

where precision (P) is the proportion of correct predicted term assignments (c) of the total number of predicted assignments (p), i.e. a measure of the accuracy of predicted terms.

R = c/t

and recall (R) is the proportion of correct predicted term assignments (c) of the total number of correct terms (t), i.e. a measure of how many of the possible correct terms were returned by the method.

Furthermore, similar to Karaoz et al [17] we have adopted the harmonic mean of the precision and recall as an overall accuracy measure. This can be defined as:

H = 2/(1/P+1/R)

where H is the harmonic mean of precision and recall for a predicted term assignment.

To examine the definition of 'a correct term' we modified the allowed edge distance between a predicted term and a curator-assigned term to assess the overall impact on accuracy scores. All UniProt submitted proteins were assigned a number of terms randomly equal to the number that had been assigned by curators. A 'correct term assignment' was defined as being when a predicted term id was within an 'allowed edge-distance' to the curator-assigned term id. The allowed edge distance between these terms was increased from equality (0) to 4, and the recall and precision calculated. This analysis demonstrated that increasing the allowed edge distance between a predicted and a curator-assigned term when deciding on which predicted terms were correct would grossly increase the perceived accuracy of annotation methods. For this reason all accuracy metrics used here required that the predicted term id and curator-assigned term id had to be equal if the predicted association was to be considered correct.

#### Annotation methods

BLAST was used to find matching proteins between the training and test sets. Two BLAST output datasets were generated: the output of matching training-set proteins to test-set proteins with an expect value cut-off of 1e^-10^, and the reciprocal search (test against training set). The command line used per query (or input) protein was similar to:

./blastall -p blastp -d blastable_db_proteins -i query-sequence -o output_file -e 1e-10

Several automated annotation methods were developed using a variety of approaches to assign terms based on BLAST output. We assigned these methods the following descriptive names based on how terms were assigned: Best BLAST, Covering Graph, Term Distance Concordance, and Discriminant Function. All methods used as input the best five BLAST-matched proteins, based on descending order of expect value, and returned a set of predicted terms, for a query-protein. Initially, work utilised all BLAST-matched proteins, but subsequent testing of these annotation methods showed that using greater than five did not result in further increases in annotation accuracy. Annotation methods were then compared using precision, recall and their harmonic mean to determine the most accurate method of automatically annotating protein sequences with GO terms. Pseudocode detailing the Covering Graph and Term Distance Concordance methods, and the Term Covariance Filter, is given in the Methods section.

#### Best BLAST method

The best matching protein sequence returned by BLAST for each input protein was selected, and terms assigned to it by curators were then assigned as predicted term for the input protein. This method is treated as the benchmark against which other methods listed below are compared.

#### Covering Graph method

The terms assigned by curators to BLAST-matched proteins for a query protein were pooled. These were then broken into groups based on term ontologies (i.e. biological process, cellular location, or molecular function). For the terms within an ontology the GO directed-acyclic-graph was examined to find the closest common-ancestor term. The paths from this term to all curator-assigned terms then defined the covering graph. All of the terms along paths within the covering graph, including the common-ancestor term and curator-assigned terms, were then assigned a 'concordance score'. This score was defined in such a way as to assign higher scores to terms that are related to a greater number of curator-assigned terms. To find this, each curator-assigned term was given a concordance score based on the expect value, and the number of times the term was associated with the best five matching proteins. This concordance score was then assigned to the term's ancestors recursively upwards in the covering graph, i.e. from the curator assigned term, then assigned to their ancestors along the covering graph, stopping when the common ancestor term is reached. A variety of methods were examined in terms of using this score to select a small number of terms. These included simply selecting the ten best scoring terms, selecting all terms with a score > 0.1, and using the Term Covariance Filter (see below). We also examined the issue of whether a maximal score cut-off could be employed to increase the accuracy of the approach. We did this by declaring a cut-off threshold value, where terms with a concordance score greater than this were excluded, and finding the accuracy score given to annotations where this was varied.

#### Term distance concordance method

The terms assigned by curators to BLAST-matched proteins for a query protein were pooled. We then defined 'term distance' to be the number of edges present in the shortest possible path between two terms. A matrix of term distances was calculated. The matrix comprised of essentially a table showing the distance of each term to every other term assigned by curators. Terms were assigned scores by calculating the sum of the product of the inverse log of the ascending expect value rank of the BLAST match and the maximum term depth divided by the distance between terms. In cases where terms were associated with more than one BLAST-matched sequence the highest ranking match was used for this calculation. Terms were selected for annotation by either selecting the 10 best ranking terms or using the Term Covariance Filter.

#### Discriminant function method

Discriminant analysis [18] was undertaken to create a model for correct term assignment. Term and result data obtained from the first five BLAST results for each query sequence were examined. Result and term data were incorporated. Results were ranked in descending order of expectation value. Duplicate terms were excluded but contributed to a count of the number of times the term appeared among the results (term-result count). Attributes included were term-result count, term depth, term usage frequency (the number of annotations using the term), the ascending rank value of the highest matching result the term was found in, BLAST score (bits) and expectation value. Two-fold cross-validation was undertaken, i.e. the data was broken into 2 groups with models built on one group and tested on the other and vice-versa. Box's M test [18] for homogeneity of covariance measures was significant. Box's M test is prone to being over sensitive for large sample sizes (N = ~16000). Log determinants were low and all attributes had high tolerance scores, indicating that covariance assumptions were not violated. Stepwise analysis indicated that all attributes were significant. Models of both training sets were significant and had highly similar discriminant functions and structure matrices, and high cross-validation accuracies (78.9% and 79.3% respectively). Due to the similarity of both models, test and training sets were combined to create an average model. This had a post hoc accuracy of 79.1%, a true negative rate of 94% and a true positive rate of 60%. The canonical discriminant function coefficients were used to score potential term associations for query sequences. Essentially terms associated with the first five BLAST matching proteins were assigned a discriminant function score. As there were only two classes to distinguish between (either a correct or incorrect prediction) those potential term associations with greater than a cut-off score (that being the Mahalanobis distance midway between the correct and incorrect points) were assigned to the sequence.

#### Term covariance filter

The Covering Graph and Term Distance Concordance annotation scoring methods outlined above have no intrinsic capacity to assign terms to protein sequences. They simply assign a score to potential term assignments that it is hoped corresponds to an increased probability that that potential term assignment is correct. Approaches to automatically assigning predicted terms to sequences were examined that utilise the scores output by these methods. As outlined above a simple method was to assign a maximum of the highest scoring ten terms to a sequence. Ten was found to be the most accurate number to assign based on the harmonic mean of the precision and recall (data not shown). A statistical approach was also examined that used a chi-square based decision-making algorithm, that we called the Term Covariance Filter. All terms returned by a scoring method were broken into groups based on ontology. All possible combinations of five or less terms were created in descending order of the sum of the scores given to their composite terms. A combination was checked against the database of term annotations to find the observed number of instances that this combination of terms had been assigned to protein sequences by curators (i.e. all term annotations that did not have an ISS evidence code). If the observed number was greater than five, then the chi-square test statistic was calculated, which was defined as:

chi-square test statistic = (o-e)^2^/e

Where o is the observed number of instances for a term combination, and e is the expected number of instances of this term combination, calculated as the product of the proportion of the total number of annotations for each term. If the chi-square test statistic was greater than the critical value (3.84), then the term combination was accepted as valid. Otherwise the next combination was examined, until only combinations consisting of a single term remained. When that occurred the best scoring term was selected.

### Analysis

#### Impact of distance on accuracy

The precision and recall measures for term assignments made by the random term assigner at different permitted distances between the predicted and correct term for a sequence were calculated. Table 1 illustrates that increasing the allowed distance between predicted and correct term results in an exponential increase in recall and precision measures, with recall more sensitive to this than precision. The relative impact on harmonic mean is the harmonic mean at a given distance divided by the harmonic mean at distance 0. The effect on the harmonic mean of the accuracy of increasing the allowed distance to 1 is to increase the accuracy value 3-fold. As a result of this all accuracy measures described in this paper use a permitted distance between predicted and correct term nodes of 0.

**Table 1:** Impact on accuracy estimates of varying allowed distance between predicted and curator-assigned terms.

**Distance**	**Relative Impact on Harmonic Mean**
0	0.00
1	3.08
2	10.32
3	40.65
4	97.06

#### Accuracy of using BLAST results for term annotation

**Figure 1:**
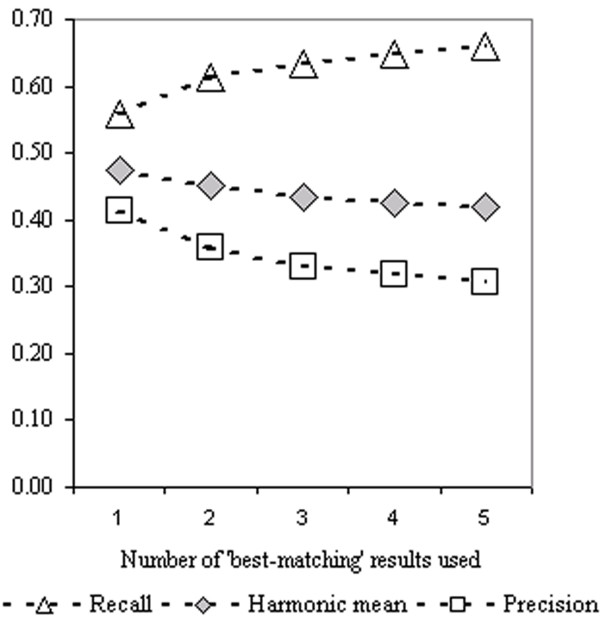
Prediction accuracy based on terms associated with a given number of best matching BLAST results.

Figure 1 demonstrates the impact on accuracy measures of increasing the number of BLAST results used for term assignment for 4,710 UniProt query sequences. Essentially as the number of BLAST results is increased the recall increases and the precision decreases. The overall impact on the harmonic mean is a slight decrease. As the number of results used increases so does the average number of term associations per query sequence (data not shown).

#### Accuracy of term assignment approaches

No combination of approaches using the Covering Graph method had a better overall harmonic mean than the Best BLAST method. Indeed the only case where any accuracy metric is higher than that of the Best BLAST method is the recall when term assignments are based on covering graph normalised concordance scores >0.1. However, this increase in recall, and respective drop in precision, was due to this method assigning approximately four times as many terms as the Best BLAST method.

The Term Distance Concordance method had a greater precision in all cases than the Covering Graph method. Where Term Covariance Filter selection was used, recall was higher than all Covering Graph annotation approaches except where assignment was made where terms had >0.1 normalised concordance. In the case of Term Distance Concordance annotations using the ten highest scoring terms, recall was higher than any Covering Graph approach. Term Covariance Filter selection increased the precision of the Term Distance Concordance method slightly while decreasing the recall by more than double this difference.

The Discriminant Function method had the best overall performance with a higher precision than all other methods including the Best BLAST method, and a recall comparable to the best recall of any other method shown. The training set was the UniProt data set used by all other methods. Compared to the other methods used, the Discriminant Function method assigned terms to less than half the total number of query sequences and assigned the least number of terms to those that it did assign terms to. This is because in many cases a query sequence had no potential term associations that had a discriminant function score greater than the cut-off. This results in a highly conservative pattern of term prediction.

## Discussion

Many approaches to GO term prediction utilise BLAST in some way. This might involve using LocusLink entries returned by online BLAST output to identify existing GO term annotations [19]. It is also common practice for researchers to use enzyme commission (EC) numbers to search for GO terms in the Kyoto Encyclopedia of Genes and Genomes database [12]. Furthermore, several recently published accounts of annotators [6,9] utilise the GO database and BLAST to find matching sequences with existing GO term annotations.

Bearing in mind that there are a great many different ways of creating a sequence function predictor based on GO and BLAST, it becomes important to demonstrate that the new system is more accurate than those already in use. Perhaps the default system of choice for use by researchers is to simply select the best matching sequence returned by BLAST that also has a GO term annotation. The advantages of this approach are speed and simplicity, biological merit in assigning function based on sequence, and that it mirrors patterns of term assignment from other existing annotations. In order to demonstrate their usefulness new approaches to assigning GO terms based on BLAST output should be benchmarked against this default approach. Unfortunately as far as the authors are aware, no other published accounts of function annotators have compared their effectiveness against simply assigning terms associated with the best matching BLAST sequence.

Annotation systems routinely address accuracy issues in an indirect or incomplete manner often using small, handpicked samples and do not demonstrate accuracy relative to techniques commonly employed by biologists. A common feature of the ad hoc manner in which accuracy has been described for some annotation systems is to increase the permitted distance between predicted and correct terms allowed before a term assignment is declared incorrect. For instance, both GOFigure and GOEngine allowed a distance of 1 between predicted and correct terms. Table 1 shows the overall impact of increasing the permitted distance between predicted and correct terms on accuracy measures for a very bad annotation method (random term assignment). At a permitted distance of 1 the precision, recall and harmonic mean are around 3 times as high as an estimate based on simple term matching. As such, accuracy estimates that do not use exact matching must be viewed sceptically. All accuracy measures used here require exact matching between terms (i.e. distance permitted is zero).

We have evaluated the accuracy of the defacto standard approach of assigning GO terms to a novel sequence based on sequence similarity to another sequence, and used this to benchmark new approaches to GO term prediction. In doing this we found that using GO terms associated with the best matching BLAST sequence for function prediction is a very effective method in and of itself. This approach is more accurate in terms of precision and recall than most of the various methods implemented here (Table 2). Furthermore, by simply increasing the number of results used the recall can be increased but with a decrease in precision.

**Table 2:** Accuracies of BLAST-based automated GO term predictors

**Method**	**Precision**	**Recall**	**Harmonic Mean**	**Mean Term Assignments**	**N**
Best BLAST	0.41	0.56	0.48	3.9	4710
Covering Graph					
Ten highest scoring terms	0.20	0.48	0.28	7.7	4710
Terms with >0.1 normalised concordance	0.19	0.61	0.29	16.1	4710
Terms with >0.1 and <0.2 normalised concordance	0.09	0.10	0.09	2.7	4710
Terms with >0.1 and <0.9 normalised concordance	0.11	0.29	0.16	7.0	4710
Term Covariance Filter selection	0.24	0.35	0.28	3.4	4710
Term Distance Concordance					
Ten highest scoring terms	0.33	0.63	0.43	6.3	4710
Term Covariance Filter selection	0.36	0.51	0.42	3.9	4710
Discriminant Function					
Training set (post hoc)	0.70	0.59	0.64	2.4	2070
Test set (apriori)	0.61	0.51	0.55	2.0	10689

Precision and recall are inextricably associated by the error rate associated with a new term association. It is a well-known property of information retrieval systems that as the recall increases the precision will decrease. Term annotation methods will have a precision and recall based on the error rate associated with assigning each new term. The probability of mistakenly assigning a term when it is actually incorrect to do so (false positive rate) will determine the precision, while the probability of rejecting a correct term association (false negative rate) will determine the recall. The challenge of developing BLAST-based GO annotation methods then becomes that of constructing an approach that has a lower false negative and false positive rate than simply choosing terms associated with the best BLAST result.

The Covering Graph method was able to associate terms to sequences even though these terms were not directly associated with matching BLAST sequences by making the assumption that ancestral terms (i.e. parent nodes to terms in the GO directed acyclic graph) could be assigned a score based on children associated with BLAST result sequences. Unfortunately the precision of this approach is generally very low, indicating a high false negative rate. In some instances its recall is higher than that of the Best BLAST method but this is achieved by increasing the average number of term associations per query sequence, and as such, the precision is very low. Note that in the case where the Covering Graph method has its highest recall (0.61), the same recall could be obtained by simply selecting terms associated with the first two best matching BLAST results with a much higher precision (precision 0.36, recall 0.61).

GoFigure [9] utilises a minimum covering graph approach to BLAST based GO term annotation. The researchers state that a significant problem in the use of a minimum covering graph approach is that as the tree is traversed upwards ancestors accumulate a higher score such that the root node (closest common ancestor) will have the highest score in all cases. To counteract this effect they employ a maximal cut-off to scores where terms with greater than this value are not included. We examined the utility of using a maximal cut-off to see whether this improved the precision and recall of term assignments. The Covering Graph method term assignments using a cut-off of 0.2 normalised concordance had a far lower precision and recall than where a cut-off of 0.9 normalised concordance was used, and this had a lower precision and recall than where no cut-off was used (i.e. a cut-off of >= 1.0). The reason for this is that when assigning proteins manual curators tended to prefer terms higher in the GO term hierarchy, i.e. closer to the root node. Utilising a cut-off threshold means that these terms are more likely to be omitted.

The Term Distance Concordance method is a term annotation system based on finding a concordance among BLAST results for GO terms. This is achieved by constructing a matrix of term-by-term distances (the number of edges between two terms). The highest scoring terms will have the largest number of siblings, ancestors and descendants in the set of terms associated with the best 5 BLAST results. Each term instance is assigned a score based on its total relatedness to other terms and the BLAST result in which it was found. As such the system is based on a number of assumptions: terms associated with better matching sequences, that appear in multiple results and have a smaller total tree distance to other terms associated with BLAST results are more likely to be correct. On face value these assumptions appear to be fairly safe, however the overall accuracy of the Term Distance Concordance method is slightly below that of simply selecting terms associated with the best BLAST result. Selecting up to the best 10 ranked terms for this approach does yield a better recall, but with a slightly poorer precision, due to annotating more terms to a sequence on average.

The Term Covariance Filter was applied to both the Covering Graph method and the Term Distance Concordance method. The advantage of this approach is that it can be applied to any BLAST-based GO term annotation scoring system, assigning the largest permuted combination of terms for a single ontology that were used by manual curators. As might be expected the overall impact of this method of selecting terms for sequences is to increase the precision by exclusion of poor term combinations while decreasing recall. In the two cases that this is applied the precision increases and recall decreases relative to simply choosing the best 10 terms. Overall the harmonic mean remains the same. As such an approach like this may be useful in cases where precision needs to be increased at the expense of recall.

The Discriminant Function method essentially assigns potential term associations a score based on a linear model of correct versus incorrect term assignments. It is a simple and easily extendible approach to utilising BLAST data for term assignment. The accuracy of the approach as demonstrated on the UniProt dataset is well in excess of that produced by the selecting terms associated with the best BLAST result. However this data was used as the training set for model fitting and as such any accuracy measures will be overestimates of that found when applied post hoc. A better estimate of model accuracy is obtained by applying the Discriminant Function method to annotate terms for all non-UniProt sequences matched against UniProt sequences using BLAST. Simply utilising the Best BLAST method for this data led to accuracy measures less than the Discriminant Function method (recall 0.55, precision 0.52, harmonic mean 0.54). Furthermore, the Discriminant Function method is far more concise than other methods, selecting fewer terms for each sequence that any other method, e.g. the Discriminant Function method selected nearly half as many terms for sequences than did the Best BLAST approach. This fact may make it invaluable to curators as a first step in a wide scale annotation project.

## Conclusion

There are a great many possible approaches to the design of systems of GO term assignment based on BLAST output, however, new approaches need to be adequately benchmarked to demonstrate their effectiveness. We have shown that an approach of simply selecting GO terms associated with the first returned BLAST matching sequence is a fairly accurate way of predicting functions for novel sequences. As such new approaches need to demonstrate that they are at least able to out perform this default approach. At the time of writing, most published accounts of functional annotators tend to provide overly generous accounts of their accuracies. To facilitate rapid developments in this area common benchmarking protocols would be useful.

We examined three approaches to GO term assignment based on BLAST output. The Covering Graph approach was able to infer associations between sequences and GO terms even though they were not directly associated with matching sequences. Unfortunately this approach has the fundamental problem where higher-level terms will always have higher scores, are more likely to be correct than lower terms, but the capacity to differentiate between correct and incorrect high level terms is lost. Maximal thresholds, where terms with a score greater than a given value are not used for annotations, are not a solution as they tend to decrease the accuracy of the system. The system preferring terms with closely related terms in higher ranked results, the Term Distance Concordance method, performed reasonably well, with accuracy measures comparable to selecting terms associated with the best BLAST result. However the approach is computationally intensive while the Best BLAST method is not. This system is probably not worth using in its current form.

A scoring system arising from discriminant analysis, the Discriminant Function method, had a higher accuracy then all methods shown here. In particular its precision was far higher than the Best BLAST method. Annotations were conservative and of high quality, and as such, this system may be of use to curators undertaking large annotation projects. This approach will be further investigated with the aim of producing a high accuracy automated functional annotation system.

In conclusion we found that accuracy benchmarking is an absolute requirement in appropriately assessing the suitability of the design of BLAST-based GO term annotators. Of the various approaches examined, a simple data-mining-oriented application is able to provide high quality, conservative functional predictions for novel sequences. To ensure that biological function annotators are of high quality, we recommend that developers utilise accuracy benchmarking and data mining techniques where possible. Future work will focus on using data mining techniques to incorporate a range of data sources to see if this increases the accuracy of function prediction.

## Methods

### Pseudocode for term prediction algorithms

A number of algorithms were developed to allow for term predictions based on BLAST-matches to protein sequences that had been assigned terms manually by curators. The more complex of these that could not be fully described in text are detailed below in pseudocode.

### Covering graph method

For each sequence

   SELECT best 5 matching sequences from results database For each term belonging to matching-sequences

      Term concordance-score = descending rank of expect value +

      number of times term found in BLAST output for this sequence.

   End for

   Construct list of unique terms from matching-sequence

   Assign matching-terms to ontology

   For each ontology

      /*Covering graph construction*/

      Find closest-common-ancestor-term

      Find all paths from ancestor term to matching terms

      Add all terms along these paths to analysis

      /*Term-score assignment*/

      For each matching-term

         While matching-term has ancestors on path to closest-common-

         ancestor-term

            Assign matching-term concordance score to ancestor

         End while

      End for

   End for

End for

### Term distance concordance method

For each sequence

   SELECT best 5 matching sequences from results database

   For each term belonging to matching-sequences

      Construct matrix to all other terms in the same ontology where

      value is the distance between terms.

      For each term in term-matrix

         Concordance score = inverse log ((ascending expect value

         rank) x (ontology tree depth/distance to matching-term)

      End for

   End for

End for

Where 'ontology tree depth' is the distance from the root to the leaves for a given ontology.

### Term covariance filter

Assign terms to ontologies

For each ontology with number of terms > 0

   If number of terms == 1

      Assign this term to sequence

   Else

      Do

         Construct array of permuted term-combinations where elements

         are ordered in descending order of combination size and sum

         of annotations, and where the maximum size of a term-

         combination is 5.

         observed = number of occurrences where term-combination [i]

         terms were all assigned to a single sequence

         While observed number < 5

            Drop lowest scoring term based on assignment method

            observed = number of occurrences where term-

            combination [i] terms were all assigned to a single

            sequence

         End while

         expected = ((n_1_/max)*(n_2_/max)... *(n_n_/max)) * max [where max

         is the total number of term observations for that ontology]

         chi-square test statistic = (observed-expected)^2^/expected

      While test statistic < chi-square critical value AND terms > 1

      Assign these terms to sequence

   End If

End for

## Authors' contributions

CEJ undertook initial study design, software implementation, statistical analysis and interpretation, and drafted the initial manuscript. UB and ALB participated in the final study design, coordinated the study and contributed to the final manuscript.
